# 553 Does COVID-19 Lead to Worse Outcomes in A Burn Center?

**DOI:** 10.1093/jbcr/irac012.181

**Published:** 2022-03-23

**Authors:** Sanja Sljivic, Lori Chrisco, Rabia Nizamani, Booker King, Felicia Williams

**Affiliations:** University of North Carolina - Chapel Hill, Chapel Hill, North Carolina; North Carolina Jaycee Burn Center, Chapel Hill, North Carolina; UNC Jaycee Burn Center, Chapel Hill, North Carolina; UNC Jaycee Burn Center, Chapel Hill, North Carolina; UNC Jaycee Burn Center, Chapel Hill, North Carolina

## Abstract

**Introduction:**

The global pandemic caused by severe acute respiratory syndrome coronavirus-2 (COVID-19) has exhausted resources and devastated at-risk populations. Our objective was to determine if COVID-positive patients have worse outcomes compared to COVID-negative patients after burn injury or desquamating skin disorders.

**Methods:**

Patients were identified using our institutional Burn Center registry and linked to the clinical and administrative data. All patients admitted between March 1, 2020 and August 31, 2021 were eligible for inclusion. Demographics, length of stay (LOS), co-morbid conditions, and mortality were evaluated. Statistical analysis was performed with Students’ t-test, chi-squared, and Fischer’s exact test.

**Results:**

A total of 1,994 patients were admitted during this period, and of those patients, 1,467 were adults. Twenty-three adults were COVID-positive. There were no significant differences in age, LOS, total body surface area (TBSA) involvement, hospital costs, sex, race or ethnicities of patients. There were no significant differences in percentage of patients presenting for burn or desquamating skin disorders. COVID-positive adult patients had a significantly higher mortality after injury than COVID-negative adults, p=0.003. There were no differences in COVID-positive pediatric patients admitted to our burn center.

**Conclusions:**

A positive COVID test is associated with worse outcomes in patients admitted for burn injury or skin-sloughing disorders. Further study is warranted to investigate and mitigate what aspect of their care could be adjusted to improve outcomes.

